# The use of Janus kinase inhibitors to treat noninfectious uveitis

**DOI:** 10.1038/s41433-025-04065-w

**Published:** 2025-11-06

**Authors:** Madeline Beckman, Sunil K. Srivastava, Careen Y. Lowder, Kim Baynes, Ashley Lowe, Sumit Sharma

**Affiliations:** 1 Cole Eye Institute, Cleveland Clinic, Cleveland, OH USA; 2 Uveitis Center of Excellence, Cleveland Clinic, Cleveland, OH USA; 3 Cleveland Clinic Lerner College of Medicine of Case Western Reserve University, Cleveland, OH USA

**Keywords:** Autoimmune diseases, Inflammation

## Abstract

**Objective:**

To present a case series of eight patients with noninfectious chronic uveitis treated at a single institution with one of two JAK inhibitors (Jakinibs), upadacitinib or tofacitinib.

**Subjects/Methods:**

A retrospective chart review of patients seen by the uveitis service from 2015 to 2022 treated with upadacitinib and/or tofacitinib was performed. All patients included had previously failed alternative immunomodulatory therapy (IMT), had consistent ophthalmic evaluations for at least 6 months, and stayed on the Jakinib for at least 3 months. Eight patients were included. Demographic information was collected. Flares were classified clinically through visual acuity, symptoms, examination, and imaging. Side effects of the Jakinibs were recorded.

**Results:**

The median length of Jakinib therapy duration was 29.5 months (range 6 months–43 months). Five patients were initiated on a Jakinib due to poor control of their ocular inflammatory diseases. Four of these patients achieved control with tofacitinib with or without additional steroid use; one patient achieved control with upadacitinib with one mild flare that resolved with systemic steroid use. Three patients started a Jakinib due to poor systemic disease control. Two of those remained flare-free of uveitis in the following year. The third patient had a flare that resolved with topical and oral therapy. Gastrointestinal upset was the most common side effect of tofacitinib. Upadacitinib had no reported side effects.

**Conclusion:**

Jakinibs may have a role in treatment of refractory noninfectious uveitis after failing conventional IMT. Both Jakinibs were well tolerated with a low incidence of side effects.

## Introduction

Chronic noninfectious uveitis can be treated with either local corticosteroid therapy or systemic immunosuppression. Adalimumab (Humira; AbbVie Inc., North Chicago, IL), a fully humanised tumour necrosis factor-α (TNF-α) antibody, was the first non-corticosteroid approved medication for the treatment of noninfectious intermediate, posterior, and panuveitis [1, 2]. Other steroid-sparing maintenance therapy options are all off label, including but not limited to the use of antimetabolites such as methotrexate and mycophenolate mofetil, calcineurin inhibitors such as cyclosporine and tacrolimus, and other biologic therapies [3].

Despite its success, 53.6% of patients had treatment failure with adalimumab at month 12 in the VISUAL-1 study [1]. A randomised control trial comparing the treatment of methotrexate to mycophenolate mofetil showed a 6 month failure rate of 25.6% and 44.7%, respectively [4]. The SITE study demonstrated that 35.7% of patients on mycophenolate mofetil and 38.3% of patients on methotrexate failed to achieve control of ocular inflammation [5]. Few randomised control studies have compared the efficacy of other steroid sparing therapies and biologics [1, 2, 6]. While biologics may have a more favourable side effect profile and utility as compared to traditional immunomodulatory therapies (IMT), their use can be limited by their potential side effects: increased susceptibility to infections, reactivation of latent viruses, hypersensitivity reactions, gastrointestinal disturbance, cardiac effects, and malignancy [7].

Despite the efficacy of currently available treatments for most patients, there remains a subset of patients who will require multiple trials of different IMTs to achieve remission. Janus kinases (JAK), a group of non-receptor tyrosine kinases, and signal transducer and activator of transcription (STAT), an intracellular receptor and transcription factor for JAK, have been implicated in the pathogenesis of inflammatory and autoimmune disorders, including rheumatoid arthritis (RA), psoriasis, Behçet’s disease, and Crohn’s disease [8–12]. A growing number of case reports have demonstrated the efficacy of JAK inhibitors (Jakinibs) in the treatment of uveitis [13–18]. Currently, the Food and Drug Administration (FDA) has approved the use of four JAK inhibitors: tofacitinib (Xeljanz; Pfizer, New York, NY), baricitinib (Olumiant; Eli Lilly/Incyte, Indianapolis, IN), upadacitinib (Rinvoq; AbbVie Inc., North Chicago, IL), and abrocitinib (Cibinqo; Pfizer, New York, NY) for various indications, including RA, psoriatic arthritis, ankylosing spondylitis, ulcerative colitis, and atopic dermatitis [19–21]. We present a cohort of patients treated with two Jakinibs, tofacitinib and upadacitinib, for noninfectious ocular inflammatory diseases and poor systemic disease control.

## Materials and methods

A retrospective chart review of patients with noninfectious uveitis was performed with IRB approval from the Cole Eye Institute, Cleveland Clinic (USA). This study adhered to the principles of the Declaration of Helsinki. Informed consent was waived due to the retrospective, non-interventional nature of this study.

This chart review included patients seen by the uveitis service (SKS, CYL, or SS) between 2015 and 2022 who were prescribed tofacitinib and upadacitinib. Patients were included in this study if they had a diagnosis of noninfectious uveitis and were treated with a Jakinib, had consistent ophthalmic evaluations for 6 or more months, and maintained at least 3 continuous months of use of the Jakinib. Patients were excluded if they did not have a diagnosis of noninfectious uveitis; if they did not have continuity with the uveitis service during their time on the Jakinib; or if they did not continue on the Jakinib for at least 3 months for reasons other than medication side effects (e.g., insurance authorisation, loss to follow-up).

Demographic information was collected, including age, sex, ethnicity, type of ocular inflammatory disease (iridocyclitis secondary to juvenile idiopathic arthritis (JIA), scleritis secondary to both JIA and RA, intermediate uveitis secondary to sarcoidosis, retinal vasculitis secondary to multifocal choroiditis (MFC), and panuveitis secondary to JIA), systemic manifestations, and duration of ocular inflammatory disease. Data collection included the dose and duration of the Jakinib therapy, the history of IMT, and the concomitant IMT with the Jakinib. Previous and concomitant therapy included oral corticosteroids, conventional immunosuppressants, biologic agents, and previous trialling of a Jakinib.

Flares were classified clinically as an overall assessment based on imaging, visual acuity, and symptoms in addition to an examination by uveitis-trained ophthalmologists. The primary outcome was defined as the number of flares in the year prior to and year after initiating the Jakinib. Side effects of the Jakinib were recorded, including but not limited to gastrointestinal symptoms, headaches, infections, malignancy, major adverse cardiovascular events (MACE), such as blood clots, myocardial infarction, or stroke, and death.

## Results

Demographic information is summarised in Table 1. Eight patients with noninfectious uveitis were included in this study. There were seven females and one male. Median age was 42 years (range 27 years–65 years). All patients were white, non-Hispanic. Half of the patients had bilateral disease (*n* = 4). Five patients had active disease prior to initiation of the Jakinib. Uveitis diagnoses include scleritis (*n* = 4), iritis (*n* = 1), intermediate uveitis (*n* = 1), retinal vasculitis (*n* = 1) and panuveitis (*n* = 1).

**Table 1:** Demographic information and clinical characteristics.

Patient	Sex/ Age	Ethnicity	Eye involvement	Type of Noninfectious Uveitis	Uveitis duration	Systemic presentation	Previous IMT	Jakinib (mg)	Duration of Jakinib	Concomitant IMT	Flares 1 y prior to switch	Flares 1 y after switch	Jakinib side effects
1	F/33	White	Both	JIA iritis	19 y	Arthropathy	MTX, AZA, PDN	Upadacitinib 15	15 mo	None	2	2^a^	None
2	F/41	White	Both	MFC	6 y	None	MMF, MTX, ADA, IFX, PDN	Tofacitinib 11	24 mo	MTX 20 mg/wk	1	0	GI upset, fatigue, rash
3	F/43	White	Both	JIA panuveitis	18 y	Arthropathy	MTX, HDX, RTX, ANA, ETN, IFX, ADA, TCZ, LEF, PDN, ABA	Tofacitinib 11	10 mo	MTX 20 mg/wk, PDN 4 mg daily	3	3	None
								Upadacitinib 15	6 mo	MTX 20 mg/wk, PDN 4 mg daily	3	1	None
4	F/27	White	Both	Sarcoidosis intermediate uveitis	5 y	None	ADA, MTX, TCZ, AZA, MMF, LEF, HDX, PDN	Tofacitinib 5	43 mo	AZA 200 mg daily	2	0	None
5	F/23	White	Right	JIA scleritis	17 y	Arthropathy	MTX, SSA	Tofacitinib 11	35 mo	None	0	0	Non
6	F/60	White	Right	RA scleritis	6 y	Arthropathy	ETN, ADA	Tofacitinib 11	43 mo	MTX 7.5 mg/wk	0	0	Morning nausea
7	M/65	White	Left	RA scleritis	5 mo	Arthropathy	ADA, MTX, ETN	Tofacitinib 11	13 mo	PDN 40 mg daily	1	1	None
8	F/63	White	Right	RA nodular scleritis	0 mo	Arthropathy	ADA, ETN, TFC	Upadacitinib 15	37 mo	None	0	1^b^	None

*ABA* abatacept, *ADA* adalimumab, *ANA* anakinra, *AZA* azathioprine, *ETN* etanercept, *HDX* hydroxychloroquine, *IMT* immunomodulatory therapy, *IFX* infliximab, *JIA* juvenile idiopathic arthritis, *LEF* leflunomide, *MTX* methotrexate, *MMF* mycophenolate mofetil, *MFC* Multifocal Choroiditis, *PDN* prednisone, *RA* Rheumatoid arthritis, *RTX* rituximab, *SSA* sulfasalazine, *TCZ* tocilizumab, *TFC* tofacitinib.

^a^Both flares occurred while patient was temporarily off of upadacitinib.

^b^Flare occurred while patient was temporarily off of upadacitinib.

Median duration of uveitis prior to starting a Jakinib was 72 months (range 5 months–19 years), excluding one patient who presented with their first episode of scleritis upon cessation of the Jakinib. Two out of the eight patients had isolated ocular inflammation, with the rest having a systemic disease associated, including JIA (*n* = 3), RA (*n* = 3), and sarcoidosis (*n* = 1). All patients in the cohort were on previous IMT prior to initiating a Jakinib, as summarised in Table 1. One patient trialled tofacitinib prior to presentation. One patient received an intravitreal dexamethasone implant unilaterally prior to Jakinib therapy to achieve control of their disease.

Five patients were switched to a Jakinib due to poor intraocular inflammatory disease control. Three patients were initiated on a Jakinib due to poor systemic disease control. Five patients received tofacitinib only, two patients received upadacitinib only, and one patient received both tofacitinib and upadacitinib during their course of treatment. Median duration of Jakinib therapy was 29.5 months (range 6 months–43 months). Five patients received concomitant therapy with their Jakinib (methotrexate, *n* = 2; methotrexate and oral prednisone, *n* = 2; oral prednisone, *n* = 1; azathioprine, *n* = 1). The remaining three patients received Jakinib monotherapy.

**Fig. 1:**
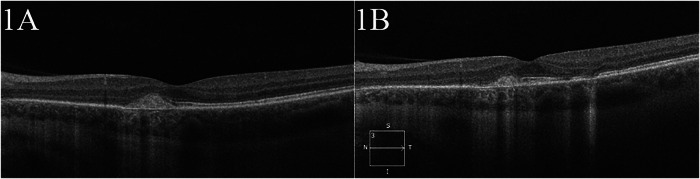
OCT of Patient 2 at presentation and at follow-up. **A** OCT of the left eye of Patient 2 at presentation demonstrating ellipsoid zone loss, choroidal neovascular membrane, and atrophy with scant intraretinal fluid. **B** Six month follow-up OCT of the left eye showing resolution of intraretinal fluid, no progression of the prior lesion but a small amount of ellipsoid loss temporally.

We now highlight three patients with different types of noninfectious uveitis refractory to previous IMT who then demonstrate sustained disease control after starting a Jakinib. Patient 2 is a 41-year-old female with a 6 year history of poorly controlled MFC. At presentation, she was taking methotrexate 20 mg weekly as monotherapy as her third-line therapy. On her initial exam at our institution, she had a drop in her visual acuity in the left eye. Optical coherence tomography (OCT) of the macula showed ellipsoid zone loss, atrophy, and choroidal neovascularisation in the left eye and scant intra-retinal fluid in both eyes (Fig. 1A). Fundus autofluorescence (FAF) and ultra-wide field fundus photos demonstrated a predominance of hypoautofluorescence with some areas of hyperautofluorescence in both eyes consistent with active MFC with new lesions. After 6 months of tofacitinib 11 mg and methotrexate 20 mg weekly dual therapy, follow-up examination and imaging demonstrated disease stabilisation with resolution of intraretinal fluid, no progression of prior lesions, but notable for temporal ellipsoid loss in the temporal fovea of the left eye (Fig. 1B). She continues to be flare-free of uveitis at 2 year follow-up.

**Fig. 2:**
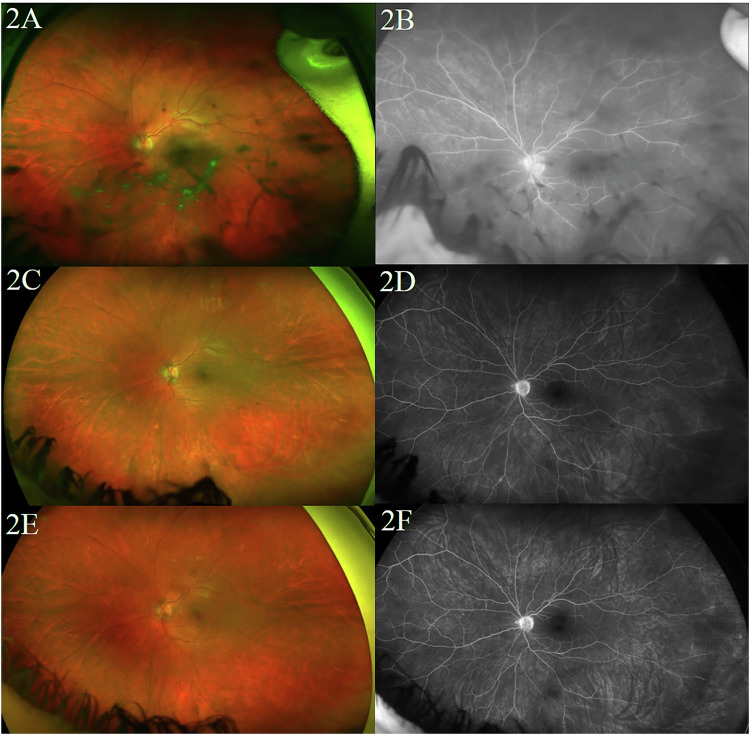
Fundus photography and FA of Patient 4 at presentation and throughout their treatment. Ultra-wide field fundus photo (**A**) and FA (**B**) of the left eye of Patient 4 prior to initiating tofacitinib demonstrating uncontrolled vitritis. Repeat interval ultra-wide field fundus photos (**C** and **E**) and FA (**D** and **F**) at 6 months demonstrating near complete resolution of leakage and complete resolution of vitritis and sustained at follow-up to 3 years after initiation of tofacitinib.

Patient 4 is a 27-year-old female with a 5 year history of sarcoid intermediate uveitis without control despite numerous medication regimens as summarised in Table 1. Ultra-wide field fundus photos and fluorescein angiography (FA) showed vitritis in both eyes, worse in left eye than the right (Fig. 2A, B). Repeat imaging at 6 month follow-up showed significantly improved peripheral leakage in both eyes (Fig. 2C, D). Three-year follow-up ultra-wide field fundus photos and FA demonstrated near complete resolution of leakage with complete resolution of vitritis (Fig. 2E, F). She remains flare-free of uveitis 4 years after initiating tofacitinib.

**Fig. 3:**
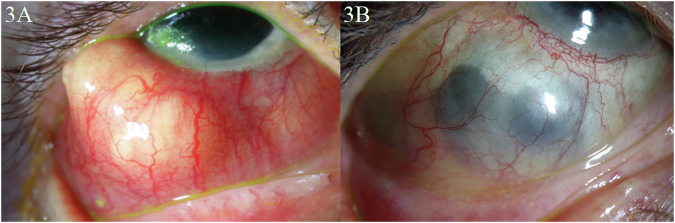
Slit lamp photography of Patient 8 at presentation and after achieving control of nodular scleritis. **A** Slit lamp photograph of the right eye of Patient 8 upon cessation of upadacitinib and initiation of etanercept demonstrating nodular scleritis. **B** Follow-up slit lamp photograph 3 months after cessation of etanercept, pulse steroid therapy, and re-initiation of her upadacitinib demonstrating near complete resolution of her nodular scleritis with remaining severe scleromalacia.

Patient 8 is a 63-year-old woman with a 15-year history of RA who had an acute episode of nodular scleritis upon cessation of upadacitinib (Fig. 3A). The patient was initially discontinued from upadacitinib due to concern for its risk of blood clots and was started on subcutaneous etanercept 50 mg weekly. After cessation of etanercept, pulse steroid therapy, and re-initiation of her upadacitinib 15 mg, she had near resolution of the nodular scleritis within 3 months, without recurrence in over 2 years of follow-up (Fig. 3B).

Tofacitinib and upadacitinib were generally well tolerated. The most common side effect of tofacitinib was gastrointestinal upset (*n* = 2). There were no reported side effects with upadacitinib. We excluded one patient who only took tofacitinib for one week due to the side effects of hypertension, headache, dizziness. This patient is not included in the discussion or the table.

## Discussion

In this case series, we report the successful use of two different Jakinibs in patients with uveitis. Included in this group are successfully controlled patients who previously failed multiple IMT regimens and continued to demonstrate persistent inflammation. All non-scleritis patients had failed multiple IMT regimens of antimetabolites and biologic agents to control their uveitis before initiating a Jakinib. Two of the four patients with scleritis were switched onto a Jakinib due to poor systemic disease control; however, they both continued to be flare-free of uveitis on tofacitinib. Two patients had uveitis flares upon temporary cessation of the Jakinib that resolved when the medication was restarted.

Tofacitinib was the first JAK inhibitor to receive FDA approval and is a preferential JAK1 and JAK3 inhibitor with some additional activity against JAK2 [22]. Tofacitinib blocks binding of IL-2, IL-4, IL-15, and IL-21 to JAK1 and JAK3, limiting lymphoid cell maturation and function [23]. Additionally, through JAK1 and JAK2 inhibition, tofacitinib decreases IFN-y, IL-6, IL-12, and IL-23 signalling. This leads to impaired differentiation and generation of CD4+ T cell helper cells and pathogenic Th17 cells, respectively [24]. Paley et al. [13] first reported the efficacy of tofacitinib in refractory idiopathic uveitis and scleritis in two patients after 4 weeks of combined treatment with methotrexate. Bauermann et al. [14]. then showed tofacitinib and methotrexate dual therapy use for JIA-associated uveitis and macular oedema, leading to both ocular and systemic disease control. Liu et al. [25]. highlighted tofacitinib’s success in reducing ocular inflammation and improving visual acuity in an 18-year-old patient with bilateral chronic idiopathic uveitis refractory to adalimumab, methotrexate, mycophenolate mofetil, and cyclosporine in combination with oral steroid therapy. In addition to uveitis, tofacitinib was shown to be successful in treatment of RA-associated ulcerative keratitis [26].

Upadacitinib is a relatively new selective JAK1 inhibitor [20]. Like tofacitinib, upadacitinib blocks IL-2, IL-4, IL-15, and IL-21 binding to JAK1 to limit lymphoid cell maturation and differentiation. Without JAK2 blocking, upadacitinib is less likely to impact hematopoiesis, as well as IL-12/23-mediated Th17 cell differentiation [23]. There are limited data on the use of upadacitinib in patients with uveitis. Baquet-Walscheild et al. [15]. published the first case report of treating JIA-associated uveitis with upadacitinib after failure of tofacitinib and methotrexate dual therapy, with a 4 month follow-up demonstrating good control of both uveitis and arthritis symptoms. Schneider et al. [16] reported on the efficacy of upadacitinib in treating HLA-B27+ anterior uveitis and macular oedema refractory to adalimumab with 8 months of follow-up. To date, no randomised control trials have been done on tofacitinib or upadacitinib for treatment of uveitis.

Other Jakinibs may also be potentially beneficial to treat uveitis. A case report of baricitinib, a selective JAK1 and JAK2 inhibitor, use in a patient with ocular and systemic features of seronegative RA showed resolution of anterior chamber cell, corneal infiltrates, vitreous opacity, and serous retinal detachment at 3 month follow-up [17, 20]. A case series on the use of baricitinib and tofacitinib for four patients with JIA-associated uveitis reported ocular inflammation control with these medications, but limited efficacy on reducing the arthritic disease burden [27]. Baricitinib use has been tested for other ocular autoimmune conditions like mucous membrane pemphigoid [28]. An open-label, Phase III clinical trial is currently underway to assess efficacy of baricitinib versus adalimumab in paediatric JIA-uveitis patients or chronic ANA-positive uveitis without extra-ocular involvement (NCT04088409). Brepocitnib is a selective JAK1 and tyrosine kinase 2 (TYK2) inhibitor that is under evaluation in a multi-centre Phase III clinical trial (NCT06431373) to determine efficacy in treatment of non-anterior non-infectious uveitis after a Phase II trial (NCT05523765) demonstrated a dose-dependent treatment benefit and was accordingly granted Fast Track Designation by the FDA.

Filgotinib (Jyseleca; Galapagos NV, Mechelen, Belgium) a preferential JAK1 inhibitor, demonstrated efficacy in reducing the risk of uveitis flare by week six of therapy in a Phase II randomised, placebo-controlled trial [18]. Filgotinib was generally well tolerated in this study with no new safety concerns identified. Unfortunately, development of this medication in the United States was halted due to business reasons. This medication was approved in 2020 to treat RA in Japan and the European Union.

Jakinib use is limited by the possible adverse reactions, most notably an increased risk for MACE such as myocardial infarction, stroke, and blood clots. This is felt to be a class effect with all Jakinibs implicated. The ORAL surveillance trial demonstrated that the non-inferiority criterion was not met for combined tofacitinib 5 mg and 10 mg regimens and TNF-α inhibitors for MACE and malignancy [29, 30]. This study included patients with RA who were 50 years of age or older, had one or more additional cardiovascular risk factor, and background treatment with methotrexate. There was also a non-dose dependent increase in malignancy, excluding nonmelanoma skin cancer, with the most frequently reported malignancy being lung cancer. A follow up study with a real-world evidence cohort suggested that the risk of MACE may be modified by baseline risk, with no association found between tofacitinib use and MACE in patients without any baseline cardiovascular risk factors [31].

The FDA and European Medicines Agency have subsequently issued warnings over the last 2 years to limit the use of Jakinibs to only patients who have an inadequate response or intolerance to one or more TNF-α inhibitors. Furthermore, special consideration must be paid to patients with the following risk factors: current or past smokers, one or more cardiovascular risk factors, development of malignancy, or known malignancy other than successfully treated nonmelanoma skin cancer [32, 33].

Despite these risks, the efficacy of Jakinibs in those who have failed other IMTs was seen in our series. Failure was defined clinically by the uveitis-trained ophthalmologists. Five patients in this case series did not have control of their uveitis in the year prior to initiating a Jakinib, ranging from one to three flares. Of those five patients, one failed to achieve control with tofacitinib; however, upon initiation of upadacitinib, they only experienced one mild flare of panuveitis that resolved with concomitant oral prednisone 4 mg daily. One patient failed to achieve ocular inflammatory control with tofacitinib monotherapy but demonstrated control after a short course of oral tapering course of prednisone starting at 40 mg. The three patients who were flare-free of uveitis in the year prior to initiating a Jakinib started the medication for poor systemic disease control. Two patients sustained ocular inflammatory control in the following year. One of these patients had a flare of scleritis after 13 months, which resolved with 1 month of fluorometholone (FML Forte; Allergan, Inc., Irvine, CA) drops and naproxen 440 mg twice a day.

Until larger trials demonstrating the safety and efficacy in uveitis patients are performed, Jakinibs will likely remain a fourth or fifth line therapy for ocular inflammatory diseases in those that are treatment refractory after corticosteroids, antimetabolites, and biologic agents. In addition to the management of isolated uveitis, Jakinibs should be considered as a fourth or fifth line therapy for the co-management of rheumatologic diseases with both systemic and ocular manifestations. Two patients were initiated on a Jakinib primarily for systemic disease control, but also benefited from having sustained ocular inflammatory control. All six patients with systemic manifestations of their autoimmune disease benefited from a reduced systemic disease burden with the Jakinib.

Limitations of the study include the retrospective, non-controlled nature of this review and the small number of participants with a short overall duration of follow-up. Furthermore, we excluded patients who did not continue the medication for more than 3 months or did not have continuity with the uveitis service, limiting the generalisability of the study.

Given the promising results in this case series and the others reported, Jakinibs may have a role for refractory ocular inflammatory disease after conventional IMT has been trialled. There was a low incidence of side effects in this patient population. However, trialling of Jakinibs must be exercised with extreme caution due to the increased risk of side effects, especially for patients with pre-existing cardiovascular risk factors. Further studies with an emphasis on randomised clinical trials will be needed.

## Summary

### What was known before

- Chronic noninfectious uveitis often requires treatment with immunomodulatory therapies (IMT).
- A subset of patients have difficult to control uveitis with current conventional IMT.

### What this study adds

- Data on the safety and efficacy of two Janus kinase inhibitors to treat noninfectious uveitis as defined as number of flares in the year prior and year after Jakinib use.
- Further evidence for Janus kinase inhibitors as a potential fourth or fifth line therapy for noninfectious uveitis.

## Data Availability

The data that support the findings in this study are not openly available due to the sensitivity of research but are available upon reasonable request to the corresponding author. Data are stored in a secure database at the Cleveland Clinic.
